# Molecular diagnosis, phylogenetic analysis, and antifungal susceptibility profiles of *Candida* species isolated from neutropenic oncological patients

**DOI:** 10.1186/s12879-023-08774-z

**Published:** 2023-11-06

**Authors:** Parviz Hassanpour, Adel Spotin, Hamid Morovati, Leili Aghebati-Maleki, Mortaza Raeisi, Mohammad Ahangarzadeh Rezaee, Alka Hasani, Ali Aghebati-Maleki, Hossein Abdollahzadeh, Sanam Nami

**Affiliations:** 1 Department of Parasitology and Mycology, Faculty of Medicine, Tabriz University of Medical Sciences, Tabriz, Iran; 2 Department of Parasitology and Mycology, School of Public Health, Tehran University of Medical Sciences, Tehran, Iran; 3 Department of Parasitology and Mycology, School of Medicine, Shiraz University of Medical Sciences, Shiraz, Iran; 4 Immunology Research Center, Tabriz University of Medical Sciences, Tabriz, Iran; 5 Hematology and Oncology Research Center, Tabriz University of Medical Sciences, Tabriz, Iran; 6 Faculty of Paramedicine, Tabriz University of Medical Sciences, Tabriz, Iran; 7 Department of Microbiology, Faculty of Medicine, Tabriz University of Medical Sciences, Tabriz, Iran; 8 Infectious and Tropical Diseases Research Center, Tabriz University of Medical Sciences, Tabriz, Iran; 9 Stem Cell Research Center, Tabriz University of Medical Sciences, Tabriz, Iran

**Keywords:** Neutropenia, *Candida* species, Molecular diagnosis, Antifungal resistance, Oncological patients

## Abstract

**Background:**

Neutropenia is the most important cause of life-threatening invasive fungal infections (IFIs). Here, we studied the frequency and antifungal susceptibility profiles of *Candida* species that colonized or caused infections among neutropenic patients with solid or hematological malignancies.

**Methods:**

A total of 362 clinical samples were collected from 138 patients. After initial isolation using a mix of mycological methods, isolates were screened using chromogenic culture media. Polymerase chain reaction-restriction fragment length polymorphism (PCR-RFLP) was applied for molecular identification. Positive or suspected cases were confirmed using the reference method of sequencing. Antifungal susceptibility testing for voriconazole and caspofungin was carried out using the microbroth dilution method. An in-silico assay was applied for phylogenetic analysis.

**Results:**

Thirty-four *Candida* strains were isolated. *C. albicans* (47.06%) and *C. glabrata* (29.41%) were the most frequent strains. Antifungal treatment reduced the chance of *Candida* colonization by almost 76% in neutropenic patients (OR: 1.759; 95% CI: 1.349 to 2.390; *p* value: 0.000). An unusual and non-resistant strain, *C. lambica*, was reported from the bloodstream of a 56-year-old man with hematologic malignancy (HM). Eight isolates were non-susceptible, and one isolate was resistant to voriconazole. Also, four isolates were non-susceptible to caspofungin.

**Conclusion:**

We can conclude that there is a cause-and-effect relationship between neutropenia, HM background, and *Candida* species separated from neutropenic patients, which can lead to possible infections. Further and repetitive studies are recommended using different molecular methods for better prediction and management of fungal infections in neutropenic patients.

## Background

Candidiasis is a vast spectrum of fungal infections caused by *Candida* yeast species [1–4]. Neutrophils are among the most prominent immune elements for host defense against invasive fungal infections (IFIs) [5]. Several studies evaluated the crucial roles played by neutrophils in defense against IFIs among immunosuppressed hosts [6, 7]. Reduced clearance by neutrophils could benefit commensal fitness and promote the virulence and pathogenicity of *Candida* species, leading to fungal positivity and infection [8, 9]. Neutropenia is the most important cause of life-threatening IFIs [5]. However, different species of bacteria also cause invasive infections in patients with prolonged neutropenia [10, 11]. Studies reported that the incidence of one of the main sorts of IFIs, nosocomial *Candida* bloodstream infections (BSIs), ranged from 0.21 episodes to 0.39 episodes per 1000 admissions among neutropenic patients [12, 13].

Clinical manifestations of IFIs among neutropenic patients are wide-spectrum and vary from simple dermatological [14] to life-threatening disseminated manifestations [15]. Due to the immunosuppressed status of the patients, the distinction between infection and colonization is very difficult [16]. IFIs require prompt diagnosis and therapy due to their life-threatening problems [17, 18]. This is complicated by the emergence of antifungal-resistant isolates, especially in *Candida* species [19, 20]. Therefore, it shouldn’t be neglected that, along with clinical signs and symptoms, microbiological and molecular diagnosis of the *Candida* species are imperative for the survival of the target population [21]. Prophylactic and empirical therapies with amphotericin B (AmB), azoles, and echinocandins are highly recommended management and control strategies [22, 23].

Here, we studied the distribution of *Candida* species among neutropenic patients with solid or hematological malignancies using molecular analysis and phylogenetic tools. Also, the antifungal susceptibility profiles of isolated strains were studied using the microbroth dilution method. This study was focused on the microbiological and epidemiological aspects of the topic.

## Materials and methods

### Patients

In our cross-sectional study, a total of 138 neutropenic patients who were hospitalized in a specialized oncology center (*Shahid Ghazi* *Tabatabai* Hospital) in Tabriz, Iran, were evaluated from December 2020 to February 2022. This study was approved by the Research Ethics Committee of Tabriz University of Medical Sciences (permission code: IR.TBZMED.REC.1399.1157). Written informed consent was obtained from patients. Moreover, medical records (malignancy type and stage, chemotherapy, and onco-radiotherapy) and demographic data (age and sex) of patients were captured and documented where available. Cancer patients with defined clinical characteristics for neutropenia [24] were included according to the oncologist recommendation. The differentiation between *Candida* positivity or negativity was first defined by the clinical signs and symptoms, such as fever, as previously described [25]. This study excluded patients with unclear pre-hospitalization, chronic fungal infection (prior to neutropenic treatment), unclear antifungal treatment (AFT) status, and unclear neutrophil count. The neutrophil count was carried out using the Sysmex KX-21 N (*Sysmex Inc*, Japan).

### Samples & initial yeast isolation

Clinical specimens, including 169 swabs from the oral cavity, 78 blood samples, 109 urine samples, and six nail samples from patients, were collected. Samples were immediately cultured on Sabouraud dextrose agar (SDA) (*Merck*, Germany) containing antibiotics (chloramphenicol) and incubated at 32 °C for 72 h. Blood samples were inoculated into biphasic brain-heart infusion (BHI) (*Baharafshan*, Iran) bottles and incubated at 37 °C for two weeks. In addition, blood specimens were inoculated into blood culture bottles (BACTEC Myco/F Lytic culture vials) and processed using the automated blood culture system (BACTEC 9120) over a 5-day incubation period. The suspected or positive cultures were transferred to sterile normal saline tubes and then immediately sent to the referral laboratory of medical mycology at the School of Medicine in Tabriz.

### Screening of *Candida* species

A loop of each tube’s content was cultured on SDA. For macro-morphologic screening of *Candida* species, the resulting single colonies were recultured linearly on chromogenic CHROMagar™ Candida plates (*HiCrome ™*, France) and incubated at 35 °C for 48 h. Also, a series of micro-morphologic tests, such as chlamydospore production on cornmeal agar (*Micro Master*, India) plus 1% Tween 80 (*Sigma*-*Aldrich*, Germany) and germ tube formation on human serum, were carried out for further evaluation. Identified colonies were purified through several dilutions and successive passages on the SDA. Pure colonies were transferred to Eppendorf tubes containing sterile water and stored at -20 °C for molecular assays and antifungal susceptibility testing (AFST).

### Molecular assays

#### DNA extraction & polymerase chain reaction (PCR)

The boiling method was applied for DNA extraction. A suspension of pure colonies was prepared in 200 µl of distilled water, boiled for 20 min in a water bath, centrifuged for 5 min at 5000 g, and the supernatant preserved at -20 °C until use [26]. The initial molecular identification was performed by PCR on the *ITS1-5.8 S-ITS2* rDNA region via ITS1/ITS4 primer pairs (ITS1: 5′-TCC GTA GGT GAA CCT GCG G-3′; ITS4: 5′-TCC TCC GCT TAT TGA TAT GC-3′) (*Sinaclon*, Iran). The PCR amplification was done in 25 µl reaction volumes, including 1 µl of each forward and reverse primer, 12.5 µl of super PCR master mix (*Yekta Tajhiz Azma*, Iran), 3 µl of DNA template, and 7.5 µl of deionized distilled water. Reactions were performed in a thermal cycler PCR system (*Peqlab*, Germany). The PCR conditions were as follows: an initial cycle of 94 ºC for 5 min, followed by 35 cycles of 94 ºC for 30 s, 56 ºC for 45 s, 72 ºC for 45 s, and a final extension of 72 ºC for 7 min. Finally, the quality of PCR products was evaluated using agarose gel electrophoresis with the Gel Doc XR system (*Biorad*, USA) and smart ladder (*Yekta Tajhiz Azma*, Iran). The positive and negative controls were 10 ng/µl of DNA from *C. albicans* ATCC 10,231 and sterile distilled water, respectively.

#### Sanger sequencing of PCR products

All positive PCR products were sent for sequencing of the amplified panfungal *ITS1-5.8 S-ITS2* gene region (*Codon genetics group*, Iran). The results of Sanger sequencing were analyzed and edited via the *Sequencher 4.7* and *MEGA 5.05* softwares. The sequences were aligned using the Basic Local Alignment Tool Search (BLAST) to check their probable similarities with submitted fungal sequences on GeneBank.

#### Polymerase chain reaction-restriction fragment length polymorphism (PCR-RFLP)

PCR-RFLP was carried out according to the previously approved protocol introduced by Mirhendi et al. [27]. However, the restriction sites were predicted by *CLC Sequence Viewer 7.6* software. The PCR products were digested by the 1 µl *Msp*I (HpaII) enzyme (*Thermo Fisher Scientific*, USA) and processed with 18 µl nuclease-free water and 2 µl 10X buffer tango [28]. Finally, *Candida* species were identified according to each electrophoretic band’s pattern and size.

#### Phylogenetic analysis

To authenticate the genetic associations and taxonomic status of identified *Candida* species, as inferred by the ITS1 and ITS4 regions, a phylogenetic tree was generated by *MEGA 5.05* software based on the Maximum Likelihood algorithm and Kimura 2-parameter model, supported by 1000 bootstrap replicates. The distance scale was estimated at 0.1. *Schizosaccharomyces pombe* (Accession number U40085.1) was considered an out-group branch. Bootstrap values higher than 70% supported the topology on each branch.

### In-vitro antifungal susceptibility testing (AFST)

AFSTs of voriconazole (VRC) (*Sigma-Aldrich*, USA) and caspofungin (CSP) (*Sigma-Aldrich*, USA) were conducted according to the Clinical and Laboratory Standards Institute (CLSI) guidelines (CLSI M27-A3) [29]. The minimum inhibitory concentration (MIC) values were interpreted according to CLSI document M27-S4 clinical breakpoints [30, 31]. The serial concentrations of the CSP and VRC were 0.016 to 16 µg/mL. For AFST, the inoculums and suspensions were prepared by the spectrophotometric method at 530 nm. The suspensions were diluted 1:1000 in RPMI 1640 medium (*Gibco*, UK) with a pH of 7.0 using 0.165 MOPS (3-N-morpholinopropane sulfonic acid) and adjusted to a final concentration of 1 × 10^3^ to 5 × 10^3^ CFU/ml. From each serial dilution, 100 µl was dispensed into columns of 96-well plates. Two columns were assigned as positive (without an antifungal agent) and negative (without fungal inoculum) controls. The plates were incubated at 35 ºC for 24 and 48 h. The 96-well plates were eventually read optically using a mirror. All tests were performed using two replicates on different days. *C. parapsilosis* (ATCC 22,019) was used for quality control in all experiments.

### Statistical analysis

The *SPSS 24.0* software was used for statistical analysis. The Kolmogorov-Smirnov and Mann-Whitney tests were used to assay the normality and mean equality of the quantitative variables (age and neutrophil count), respectively. The Chi-square method was used to compare the qualitative data (gender, AFT, and malignancy type). Moreover, the Spearman test was applied to evaluate the correlation between quantitative variables. We did not apply the Pearson correlation due to the fact that our quantitative variables did not have a normal distribution. Statistical significance was defined as *p* values less than 0.05.

## Results

### Patients characteristics

**Fig. 1:**
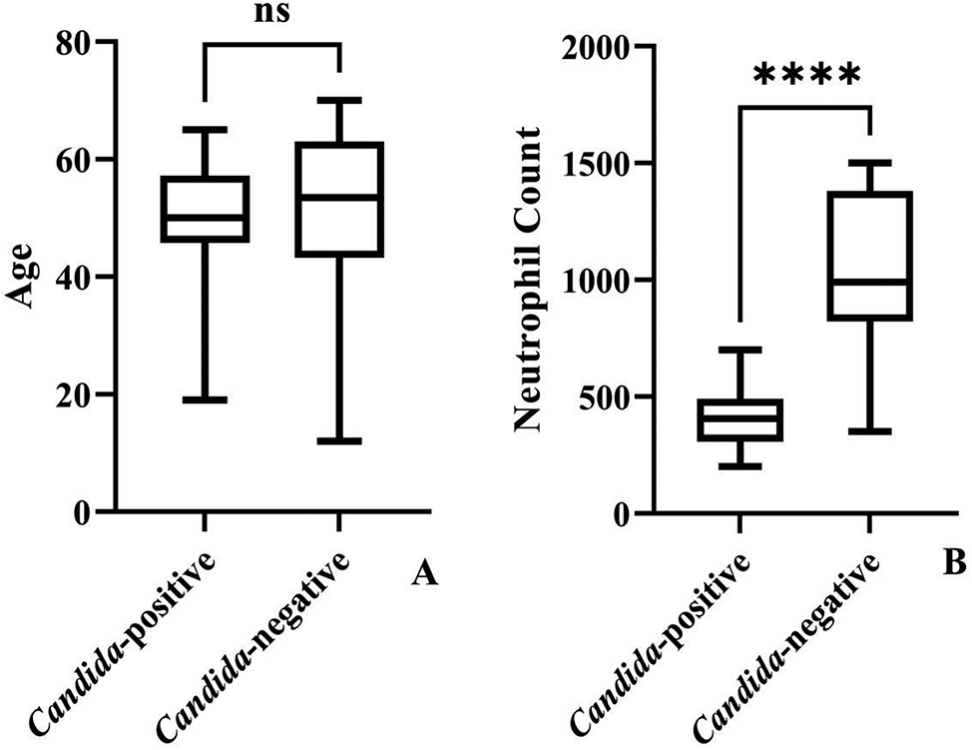
Quantitative distribution of age and neutrophil levels

A total of 362 clinical samples were isolated from 138 neutropenic patients. Thirty-four were *Candida*-positive (24.63%). Patients ranged in age from 12 to 65 years old. *Candida*-positive patients had a median age of 36 (19–65) years old, and the median age of *Candida*-negative patients was 53.5 (12–70) years old (Table 1). According to the Mann-Whitney test, the ages of the patients in the two groups were statistically similar (*p* value: 0.452) (Fig. 1A).

**Table 1:** The analysis results of quantitative variables of the study

Quantitative variable	Group	Patients	Mean/Median	95% CI/ STD deviation	Test of Normality via Kolmogorov-Smirnov	Mann-Whitney
Age	*Candida*-positive	34	Mean: 51.14 Median: 50.00	95% CI: 47.85 to 54.43 STD dev: 9.43	*p* value: 0.000	*p* value: 0.452
	*Candida*-negative	104	Mean: 52.01 Median: 53.50	95% CI: 49.75 to 54.28 STD dev: 11.62		
Neutrophil	*Candida*-positive	34	Mean: 418.088 Median: 405.000	95% CI: 373.874 to 462.302 STD dev: 126.718 Min: 200; Max: 700	*p* value: 0.000	*p* value: 0.000
	*Candida*-negative	104	Mean: 1052.884 Median: 990.000	95% CI: 991.240 to 1114.528 STD dev: 316.977 Min: 350; Max: 1500		

**Fig. 2:**
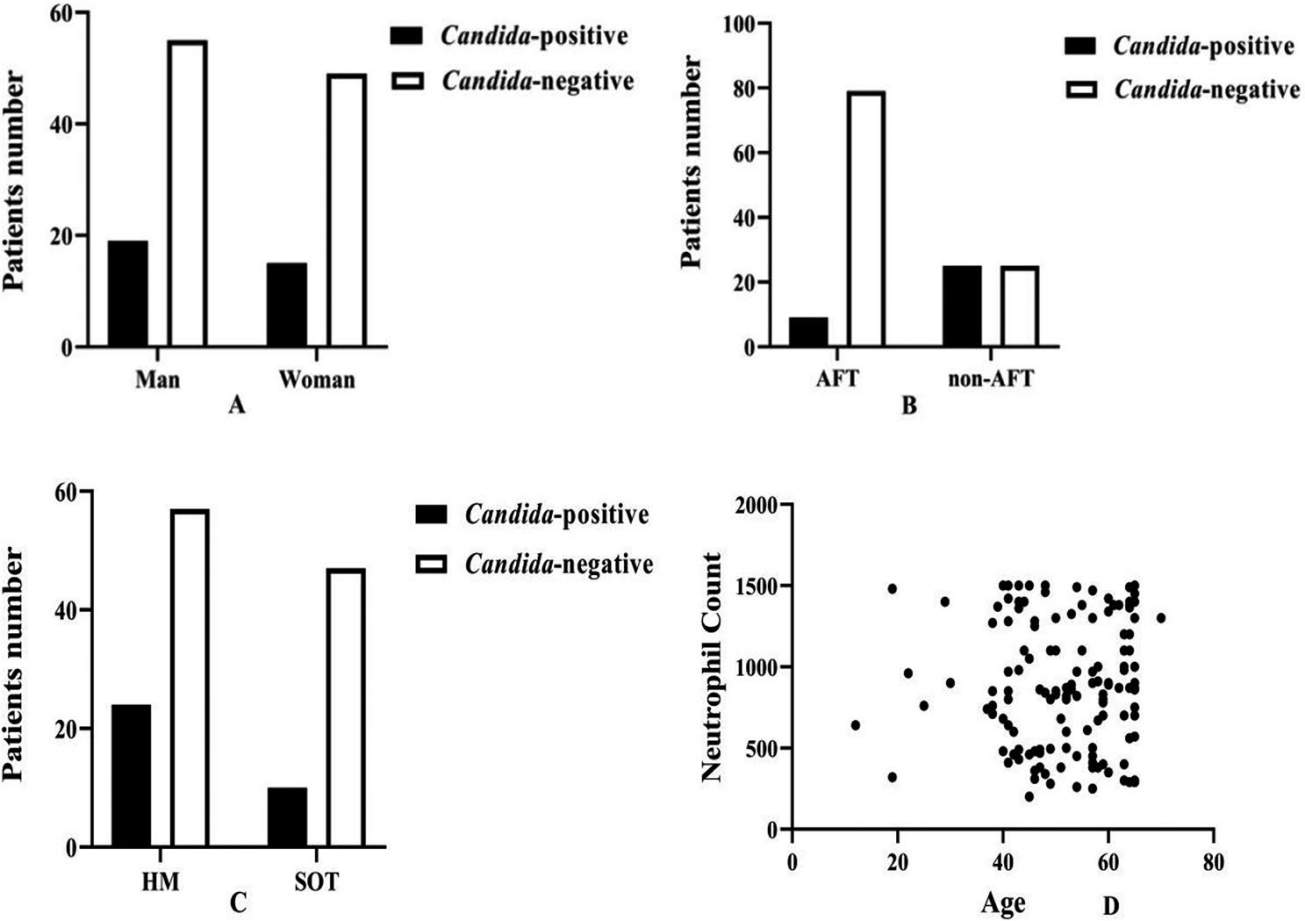
Column charts of sex ***(A)***, antifungal therapy ***(B)***, underlying cancer ***(C)***, and scatter chart of correlation between patient’s age and neutrophil counts ***(D)***

Nineteen (25.7%) *Candida*-positive patients and 55 (74.3%) *Candida*-negative patients were men. The chi-square test indicated that gender was equally distributed among the two groups (*p* value: 0.844). Moreover, we found that men have a 22% higher chance rate for *Candida* positivity than women (odds ratio; OR: 1.22; 95% confidence interval; CI: 0.519 to 2.421). However, this was statistically insignificant (*p* value: 0.844) (Table 2 and Fig. 2A).

**Table 2:** The results of analysis for qualitative variables

Variable		*Candida*-positive	*Candida*-negative	Chi-square statistics	Odds-ratio
Gender	Men	19 (25.7%)	55 (74.3%)	Pearson: 0.093 *p* value: 0.844	OR: 1.22 95% CI: 0.519 to 2.421 *p* value: 0.844
	Women	15 (23.4%)	49 (76.6%)		
AFT	Positive	9 (10.2%)	79 (89.8%)	Pearson: 27.164 *p* value: 0.00	OR: 1.759 95% CI: 1.349 to 2.390 *p* value: 0.000
	Negative	25 (50.0%)	25 (50.0%)		
Underlying condition	HM	24 (29.6%)	57 (70.4%)	Pearson: 2.632 *p* value: 0.114	OR: 1.979 95% CI: 1.113 to 4.550 *p* value: 0.002
	nHM	10(17.5%)	47 (82.5%)		

HM: hematological malignancy, nHM: non- hematological malignancy

Seventy-nine *Candida*-negative patients had received AFT, but only nine *Candida*-positive patients had received it. This difference was statistically significant (*p* value: 0.00). AFT reduced the chance of *Candida* positivity by almost 76% in neutropenic patients (OR: 1.759; 95% CI: 1.349 to 2.390; *p* value: 0.000) (Fig. 2B and Table 2).

Among *Candida*-positive patients, 24 (70.59%) had a hematologic malignancy (HM) background, and 10 (29.41%) had a solid tumor (ST) background. Furthermore, the HM background increased the chance of *Candida* positivity by 98% when compared to the ST background (OR: 1.979; 95% CI: 1.113 to 4.550; *p* value: 0.002) (Fig. 2C and Table 2).

**Fig. 3:**
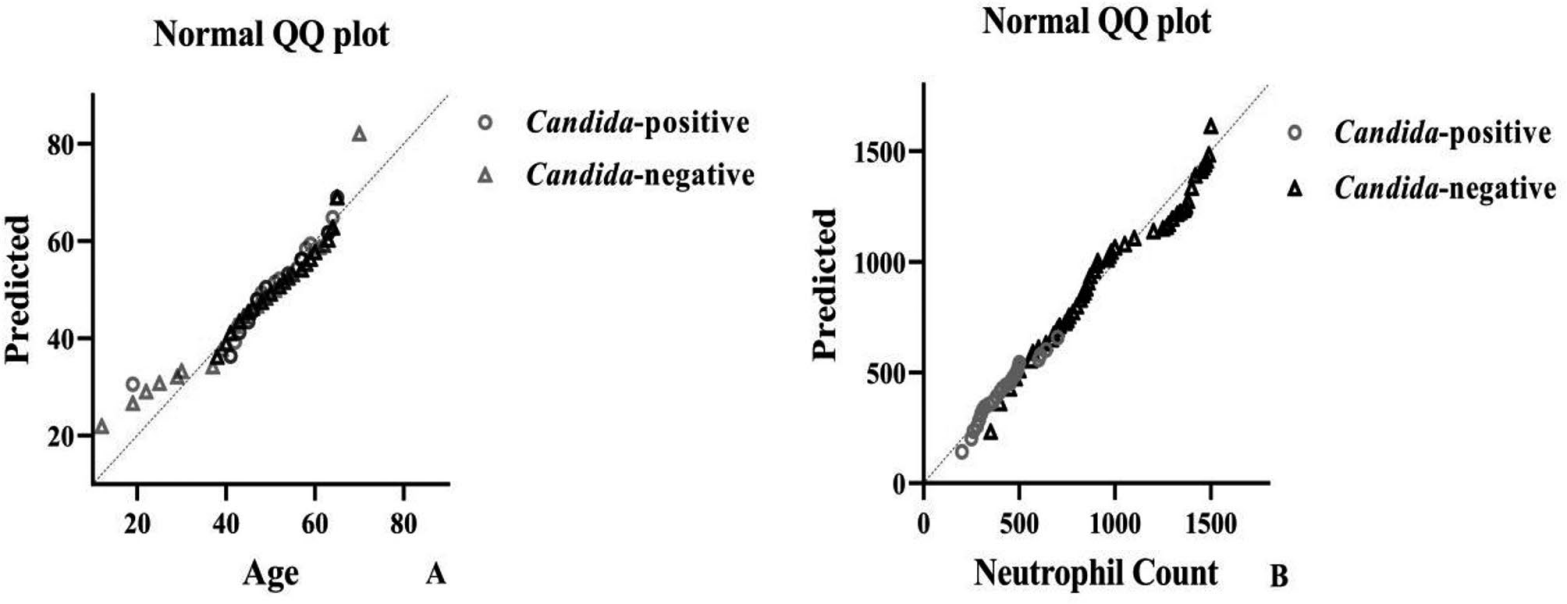
Normal distribution plots for quantitative variables of the study

Moreover, we indicated that there was no statistical relationship between patients’ age and the severity of neutropenia (neutrophil count) (Spearman’s ratio: 1.00; *p* value: 0.637) (Fig. 2D). The median levels of neutrophil count among *Candida*-positive patients were 405 (200–700) cells/µl and among non-*Candida* patients, 990 (220–989) cells/µl. Also, the Mann-Whitney test indicated that the median neutrophil count differed statistically between *Candida*-positive and *Candida*-negative patients (*p* value: 0.00) (Fig. 1B and Table 1). According to Kruskal-Wallis test results, in this study, quantitative variables were not distributed normally (Fig. 3A and B, and Table 1).

### Microbiological identification of yeasts

A total of 34 yeasts among 362 clinical samples from 138 neutropenic patients were collected and identified according to culture results on SDA and CHROMagar™ Candida medium. The isolates were as follows: 16 *C. albicans*, 10 *C. glabrata*, 4 *C. krusei*, 2 *C. parapsilosis*, and 1 *C. tropicalis.* Six isolates were suspected to be *Candida* species, which were considered for further evaluation by molecular methods. Moreover, all 16 *C. albicans* isolates were positive for chlamydospore production and germ tube formation examinations (Table 3). Thirteen isolates (38.23%), including 6 *C. albicans*, 5 *C. glabrata*, 1 *C. parapsilosis*, and 1 *C. krusei*, were collected from the oral cavity. Twelve isolates (32.25%), including 6 *C. albicans*, 3 *C. glabrata*, 1 *C. parapsilosis*, and 2 *C. krusei*, were isolated from the bloodstream. Six isolates (18.75%), including 3 *C. albicans*, 2 *C. glabrata*, and 1 *C. tropicalis*, were isolated from the urinary tract stream. Three isolates (8.82%), each one of which was *C. albicans*, *C. glabrata*, and *C. krusei*, were isolated from nail ulcers. Also, the six unknown isolates were collected from the oral cavity, blood, and urinary tract streams.

**Table 3:** Summary of findings of two diagnostic methods and sequencing confirmation

Yeast species	CHROMagar™ Candida N (%)	RFLP			Reference sequencing via SANGER *
		Frequency (N: 34)	Size of PCR Amplicon (bp)	Size of *Msp*I -RFLP fragments (bp)	
*C. albicans*	16 (47.05%)	16 (47.05%)	537	239, 298	16 (47.05%) [OP647129.1 OP647128.1 OP647127.1]
*C. glabrata*	10 (29.41%)	10 (29.41%)	881	320, 561	10 (29.41%) [OP650915.1]
*C. krusei*	4 (12.39%)	4 (12.39%)	510	250, 260	4 (12.39%) [OP647217.1]
*C. parapsilosis*	NA	2 (5.88%)	530	530	2 (5.88%) [OP658936.1 OP658946.1]
*C. tropicalis*	1 (2.94%)	1 (2.94%)	526	186, 340	1 (2.94%) [OP658947.1]
*C. lambica*	NA	NA	NA	NA	1 (2.94%) [OP658919.1]

*: Due to avoid extra content, all sequences for *C. albicans*, *C. glabrata*, and *C. krusei* were not deposited on the GeneBank

NA: not applicable

### Yeast identification based on the RFLP-PCR and sequencing reference method

**Fig. 4:**
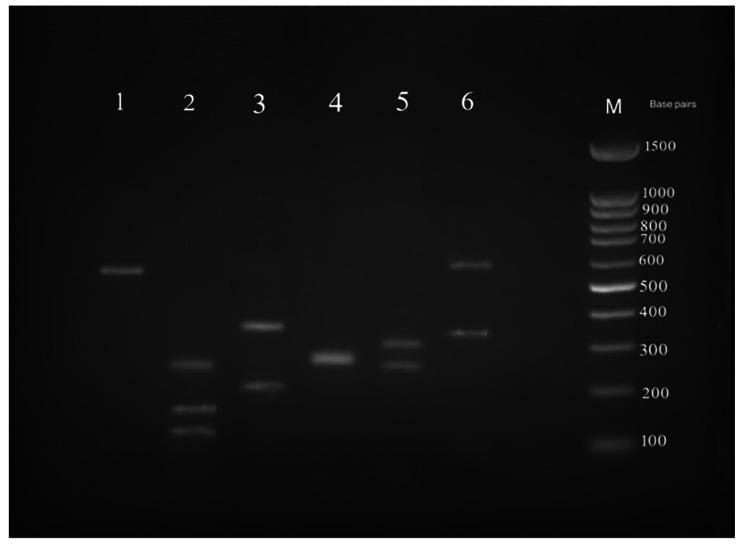
Agarose gel electrophoresis of restriction digestion by the *MspI* enzyme of *Candida* strains in PCR- RFLP; lanes 1–6. lane 1, *C. parapsilosis*; lane 2, *C. lambica*; lane 3, *C. tropicalis*; lane 4, *C. krusei*; lane 5, *C. albicanse*; lane 6, *C. glabrata.* M lane is 100 bp DNA markers

All positive and suspected isolates were considered for further evaluation via RFLP-PCR. All *Candida* species that are identified via microbiological methods were confirmed in the RFLP-PCR assay (Fig. 4 and Table 3). Among the six suspected isolates, five were negative and excluded, and one was positive but undistinguishable on the electrophoresis. Online alignment and comparison of the ITS sequencing results for the PCR products of all 33 positive and 1 suspected isolates confirmed the RFLP-PCR findings. Furthermore, ITS sequencing confirmed that one suspected isolate was the rare *C. lambica* species, which was isolated from the bloodstream of a 56-year-old neutropenic man with an HM background. The results of molecular assays for 34 *Candida* isolates and GeneBank accession numbers are presented in Table 3; Fig. 4.

### Phylogenetic analysis

**Fig. 5:**
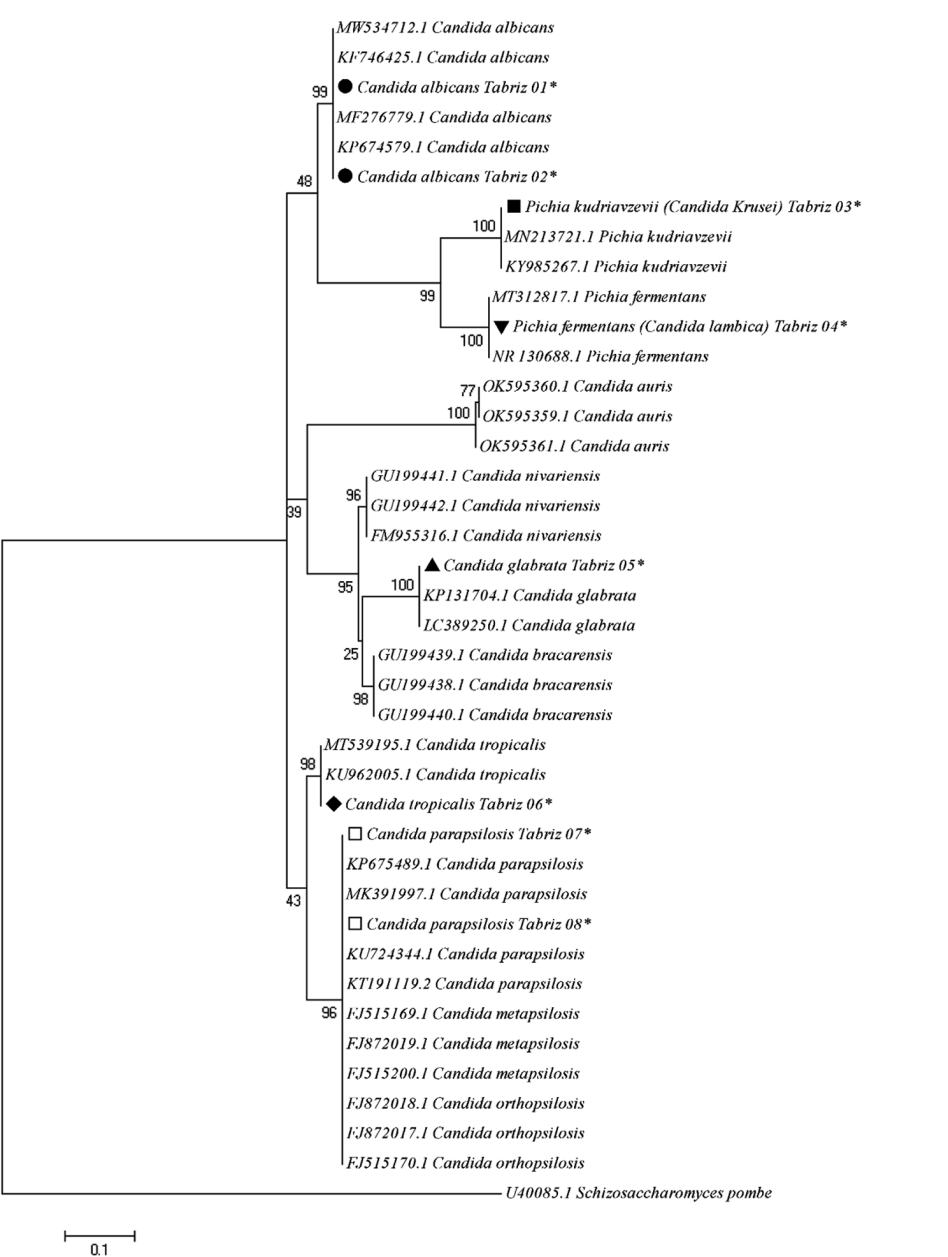
The Phylogenetic tree based on *ITS1 and ITS4* gene regions of *Candida* spp. using kimura 2-parameter model in Maximum Likelihood algorithm with 1000 bootstrap re-sampling. *Schizosaccharomyces pombe* (Accession number, U40085.1) was considered as an out-group branch. Bootstrap values of higher than 70% were supported the topology on each branch. Registered sequences characterized by asterisk (*) in this study

The topology of identified *Candida* species using ITS1 and ITS4 regions indicated that *C. albicans*, *C. krusei, C. lambica, C. glabrata*, *C. tropicalis*, and *C. parapsilosis* were placed in their specific clades (Fig. 5; marked by an asterisk [*]). Cladistic trees indicated that the *Pichia kudriavzevii* (*C. krusei*) clade has a sister relationship with the *Pichia fermentans* (*C. lambica*) clade.

### Antifungal susceptibility testing (AFST)

According to the AFST results for VRC and CSP, 8 *Candida* isolates were non-susceptible, and 1 isolate was resistant to VRC and was isolated from the urinary tract stream of a woman with a HM background. Also, four isolates were not susceptible to CSP. Tables 4 and 5 show the MIC and epidemiological cut-off values, as well as susceptibility profiles for all *Candida* isolates and tested antifungals. The MIC range for CSP is 0.015 to 4 µg/mL; the MIC50 is 0.06 µg/mL; the MIC90 range is 0.133 to 0.375 µg/mL; and the MIC range for VRC is 0.03 to 2 µg/mL; the MIC50 is 0.12 to 0.185 µg/mL; and the MIC90 is 0.133 to 0.550 µg/mL.

**Table 4:** MIC profiles of antifungal susceptibility testing (AFST).

*Candida* spp	Antifungal	MIC Range	GM	MIC50	MIC90	MIC (µg/mL) [n]										
						16	8	4	2	1	0.5	0.25	0.12	0.06	0.03	0.015
* C. albicans* (n = 16)	CSP	0.015 − 0.5	0.082	0.06	0.375	NA	0	0	0	0	2	2	2	6	3	1
	VRC	0.03-2.0	0.116	0.12	0.375	0	0	0	1	0	1	2	5	5	2	NA
*C. glabrata* (n = 10)	CSP	0.015–0.25	0.064	0.06	0.133	NA	0	0	0	0	0	1	3	3	2	1
	VRC	0.06-1.0	0.173	0.185	0.55	0	0	0	0	1	2	2	1	4	0	NA
*C. krusei* (n = 4)	CSP	0.03–0.06	0.05			NA	0	0	0	0	0	0	0	3	1	0
	VRC	0.25-1	0.294			0	0	0	0	2	1	1	0	0	0	NA
*C. parapsilosis* (n = 2)	CSP	1–4	2			NA	0	1	0	1	0	0	0	0	0	0
	VRC	0.12–0.25	0.173			0	0	0	0	0	0	1	1	0	0	NA
*C. tropicalis* (n = 1)	CSP	0.12	0.12			NA	0	0	0	0	0	0	1	0	0	0
	VRC	0.5	0.5			0	0	0	0	0	1	0	0	0	0	NA
*C. lambica* (n = 1)	CSP	0.12	0.12			NA	0	0	0	0	0	0	1	0	0	0
	VRC	0.5	0.5			0	0	0	0	0	1	0	0	0	0	NA

MIC: minimum inhibitory concentration, GM: geometric mean, CSP: caspofungin, VRC: voriconazole, NA: not applicable

**Table 5:** Epidemiological cut-off value of antifungals and susceptibility profiles of isolated *Candida* species

*Candida* species	VRC						CSP					
	ECV Profile			Susceptibility Profile			ECV Profile			Susceptibility Profile		
	ECV	≤ECV	>ECV	S	SDD	R	ECV	≤ECV	>ECV	S	I	R
*C. albicans* (n = 16)	0.06	7	9	12	3	1	0.25	14	2	14	2	0
*C. glabrata* (n = 10)	0.25	7	3	- ^*^	-^*^	- ^*^	0.5	10	0	9	1	0
*C. krusei* (n = 4)	0.5	2	2	2	2	0	0.5	4	0	4	0	0
*C. parapsilosis* (n = 2)	0.03	0	2	0	2	0	4	2	0	1	1	0
*C. tropicalis* (n = 1)	0.12	0	1	0	1	0	0.5	1	0	1	0	0
*C. lambica (n = 1)*	0.12	0	1	1^*^	0	0	0.06	0	1	1^*^	0	0

VRC: voriconazole, CSP: caspofungin, ECV: epidemiological cut-off value, R: resistant, S: susceptible, SDD: susceptible dose-dependent, I: intermediate

^*^Breakpoints not provided by CLSI document M27-S4.

## Discussion

Candidiasis is one of the most prevalent infections among neutropenic patients. There are several studies that report the predominant *C. albicans* species among different immunosuppressed patients, especially neutropenic patients. Al Hatmi et al. [32], Jafarian et al. [33], and Brescini et al. [34] reported 26.7%, 36%, and 51% prevalence rates for *C. albicans*, respectively, while Hamzavi et al. [35] reported 72%. Al Hatmi et al. [32] discovered *C. glabrata* to be the most common non-*albicans* isolate (22.7%), as we did. Despite our findings, Hamzavi et al. [35] didn’t find any significant relationship between *Candida* colonization and age, sex, oncologic diseases, or degree of neutropenia. However, Villanueva et al. [36] reported that *C. albicans* was the most predominant agent of IFI in non-neutropenic patients with solid tumors, while *C. tropicalis* was the most predominant agent in neutropenic patients with hematological neoplasia. Therefore, we can conclude that it seems weird that *C. glabrata* was the predominant non-*albicans* strain in our study. However, we were not able to recognize IFIs due to limited access to patient’s data.

Husni et al. [37] reported conflicting results compared to Villanueva et al. [36]. They concluded that the balance of *C. albicans* and non-*albicans* strains corresponding to colonization or infection in neutropenic and non-neutropenic populations is not different [37]. However, there are several reasons to be concerned about the increase of non-*albicans* strains, which can be important for their resistance to antifungal issues. Some studies reported *C. parapsilosis* [38, 39], and some others [12, 40] reported *C. tropicalis* as the most frequent non-*albicans* strain in their neutropenic papulations. Also, Kronen et al. [41] reported a high hazard ratio (HR: 1.297, 0.909 to 1.849) for 59 *C. krusei* isolates that accounted for 64.4% of death episodesamong 1873 patients. For various reasons, we can conclude that an isolation site cannot be a reliable guide for predicting the pathogenicity of *Candida* species. One main reason, which was also seen in our findings, is the mutual source of *Candida* isolates [42]. In support of our findings, we found a study [43] that reported that urinary tract BSI was the source of 20.8% of overall BSI isolates in neutropenic patients.

Our findings show that *Candida*-positive patients had a median age of 36 (19–65) years, of whom 19 (25.7%) were men (*p* value: 0.00). We found that men have a 22% higher chance rate for *Candida* positivity than women (OR: 1.22; 95% CI: 0.519 to 2.421). However, this was statistically insignificant (*p* value: 0.844). In Al-Hatmi et al.’s study [32], the mean age of the positive patients was 54 years old, and 55.4% of them were men. Similarly, Brescini et al. [34] reported that the majority of their positive patients were males with a median age of 68 years old. However, the mean age of patients in Jafarian et al.’s study [33] was 7.85 years old due to their target population and inclusion criteria. Although we reached the insignificant statistical findings, these comparisons support our claim that neutropenic men are more susceptible to *Candida* positivity; however, the age of the patients is affected by the underlying diseases and the severity of their therapeutic strategies.

In the context of antifungal therapy, our findings were supported by the Zheng et al. study [13], which reached a success rate of 76.1% for AFT. Several studies have discussed AFT of neutropenic patients and reached acceptable findings [37, 38, 40, 44–47]. Cuervo et al. [48] suggested that the ESCMID and IDSA guidelines for antifungal therapy in neutropenic patients with candidemia be used. Despite several reports of unfavorable outcomes for some antifungals, Chandrasekar et al. [49] indicated a lower success rate for the clinical administration of micafungin in neutropenic patients. However, we can conclude that AFT was successful and should be considered in empirical and prophylactic therapy schemes for better management of neutropenic patients with risk of *Candida* positivity or infection.

In our study, 8 of 34 *Candida* isolates (23.53%) were non-susceptible, and one isolate (2.63%) was resistant to voriconazole. This isolate was isolated from the urinary tract stream of a woman with an HM background. Also, four isolates (11.76%) were not susceptible to CSP. During the six-year study, Bansal et al. [45] reported an anti-azole resistance rate of 20.0% (23/115) with a significant increase for *C. tropicalis*, which is similar to our findings. Also, Kimura et al. [50] indicated that 80% of the *Candida* strains isolated from patients with HMs and neutropenia were susceptible or showed wild-type susceptibility (72%) to micafungin. Also, Liu et al. [51] reported that 33.9% of isolates were non-susceptible to fluconazole (MIC50, 2 mg/L; MIC90, 16 mg/L; MIC range, 0.25 to > 256 mg/L), while 56.9% were non-susceptible to voriconazole (MIC50, 0.25 mg/L; MIC90, 1 mg/L; MIC range, 0.015 to > 8 mg/L) according to CLSI clinical breakpoints. We can conclude that the resistance patterns of our isolated species are not different from previous studies’ findings and don’t add novel findings to the context.

Despite the fact that we did not report any non-*albicans*-resistant strains, researchers came to conflicting conclusions. Ko et al. [52] indicated a high hazard ratio of fluconazole-resistant *C. glabrata* isolates causing BSI among neutropenic patients (hazard ratio; HR 3.960, 95% CI 1.395–11.246, *p* value: 0.010). Kim et al. [53] indicated an increased rate of candidemia and a sharp switch to fluconazole-resistant non-*albicans* species in Korea. In line with our findings, Breda et al. [54] reported that in neutropenic patients with candidemia, *C. albicans* isolates were more susceptible to antifungals than non-*albicans* isolates.

When compared to ST, the HM background increased the chance of *Candida* positivity by 98% (OR: 1.979; 95% CI: 1.113 to 4.550; *p* value: 0.002). We found that the median neutrophil count differed statistically between *Candida*-positive (405 [200–700] cells/µl) and *Candida*-negative (990 [220–989] cells/µl) patients (*p* value: 0.00). There are several studies that support our findings [40, 41, 43, 45, 53, 55–59].

Concerns have been raised about the emergence of uncommon *Candida* species strains [60], particularly in neutropenic patients. We discovered an unusual strain, *C. lambica*, in the bloodstream of a 56-year-old man with HM. Fortunately, the AFST revealed no resistance status against either of the tested antifungals (MIC for VRC: 1 µg/mL and MIC for CSP: 0,12 µg/mL). Vervaeke et al. [61] reported a pathogenic case of *C. lambica* in the bloodstream of an intravenous drug abuser, a 19-year-old man. In comparison, we discovered similar susceptibility profiles to VRC. Vervaeke et al. [61] reached susceptible MIC values of 0.125, 0.064, and 2 µg/mL for AmB, ITC, and flucytosine, respectively, and a resistant MIC value of > 64 µg/mL for FLC.

## Conclusion

We concluded that neutropenia and hematological malignancies were the main risk factors for *Candida* isolation from cancer/neutropenic patients. Also, the prevalence of non-*albicans* strains remained high in this population. Antifungal therapy was the most effective inhibitor of positivity of *Candida* species. This positivity can lead to the possible infection under specific circumstances. As a result, we recommend following infection control guidelines. This study focused on the microbiological and epidemiological characteristics of *Candida* species among neutropenic patients. Further studies utilizing various molecular diagnostic methods and antifungal susceptibility testing over different time periods are recommended for better prediction, investigating the clinical findings, and management of fungal infections in neutropenic patients.

## Data Availability

The six sequences generated in this study were deposited in the GenBank database (accession numbers with persistent accessible links of: OP647129.1, OP647128.1, OP647127.1, OP650915.1, OP647217.1, OP658936.1, OP658946.1, OP658947.1, OP658919.1. All data generated or analyzed during this study are included in this published article and its related links.
