# Correction: Molecular diagnosis, phylogenetic analysis, and antifungal susceptibility profiles of Candida species isolated from neutropenic oncological patients

**DOI:** 10.1186/s12879-024-09053-1

**Published:** 2024-02-13

**Authors:** Parviz Hassanpour, Adel Spotin, Hamid Morovati, Leili Aghebati-Maleki, Mortaza Raeisi, Mohammad Ahangarzadeh Rezaee, Alka Hasani, Ali Aghebati-Maleki, Hossein Abdollahzadeh, Sanam Nami

**Affiliations:** 1 Department of Parasitology and Mycology, Faculty of Medicine, Tabriz University of Medical Sciences, Tabriz, Iran; 2 Department of Parasitology and Mycology, School of Public Health, Tehran University of Medical Sciences, Tehran, Iran; 3 Department of Parasitology and Mycology, School of Medicine, Shiraz University of Medical Sciences, Shiraz, Iran; 4 Immunology Research Center, Tabriz University of Medical Sciences, Tabriz, Iran; 5 Hematology and Oncology Research Center, Tabriz University of Medical Sciences, Tabriz, Iran; 6 Faculty of Paramedicine, Tabriz University of Medical Sciences, Tabriz, Iran; 7 Department of Microbiology, Faculty of Medicine, Tabriz University of Medical Sciences, Tabriz, Iran; 8 Infectious and Tropical Diseases Research Center, Tabriz University of Medical Sciences, Tabriz, Iran; 9 Stem Cell Research Center, Tabriz University of Medical Sciences, Tabriz, Iran

Following publication of the original article [1], we have been notified that “Infectious and Tropical Diseases Research Center, Tabriz University of Medical Sciences, Tabriz, Iran” should be shown as Prof. Sanam Nami’s first affiliation instead of “Department of Parasitology and Mycology, Faculty of Medicine, Tabriz University of Medical Sciences, Tabriz, Iran”.

The original article has been corrected.
